# Obg-like ATPase 1 inhibited oral carcinoma cell metastasis through TGFβ/SMAD2 axis in vitro

**DOI:** 10.1186/s12860-020-00311-z

**Published:** 2020-09-14

**Authors:** Jianzhou Liu, Qing Yang, Kevin Chen Xiao, Thomas Dobleman, Shen Hu, Gary Guishan Xiao

**Affiliations:** 1 State Key Laboratory of Fine Chemicals, Department of Pharmaceutical Sciences, School of Chemical Engineering, Dalian University of Technology, Dalian, 116024 China; 2 School of Bioengineering, Dalian University of Technology, Dalian, 116024 China; 3 School of Dentistry, University of California Los Angeles, Los Angeles, 90095 USA; 4 Functional Genomics and Proteomics Center, Creighton University Medical Center, Omaha, 68131 USA

**Keywords:** Metastasis, OLA1, EMT, Oral squamous cell carcinoma, SMAD2

## Abstract

**Background:**

The human Obg-like ATPase 1 (OLA1) protein has been reported to play an important role in cancer cell proliferation. The molecular mechanism underlying OLA1 regulated oral metastasis is still unknown. We investigated in this study the regulatory role of OLA1 playing in oral squamous cell metastasis.

**Results:**

A series of in vitro assays were performed in the cells with RNAi-mediated knockdown or overexpression to expound the regulatory function of OLA1 in oral cancer. We found that the endogenous level of OLA1 in a highly metastatic oral squamous cell line was significantly lower than that in low metastatic oral cells as well as in normal oral cells. Escalated expression of OLA1 resulted in a reduced ability of metastasis in highly metastatic cells, and enhanced its sensitivity to the paclitaxel treatment. Further analysis of the EMT markers showed that Snail, Slug, N-cadherin were up-expressed significantly. Meanwhile, E-cadherin was significantly down-regulated in the oral cancer cells with OLA1-knocked down, suggesting that OLA1 inactivated EMT process. Furthermore, we found that OLA1 suppressed oral squamous cell metastasis by suppressing the activity of a TGFβ/SMAD2/EMT pathway.

**Conclusion:**

Our data suggests that OLA1 may be developed as a potential target for the treatment of oral cancer metastasis.

## Background

Oral squamous cell carcinoma (OSCC) is the leading cause of morbidity and mortality in patients with head and neck squamous cell cancer (HNSC). The five-year survival rate for Oral cell carcinoma (OCC) is only about 50% [1, 2]. While, OSCC is a typical oral malignancy ranking at the sixth among all cancer death worldwide, and accounts for 90% of the incidence of OCC [3]. Although the incidence rate has decreased, the outcome has stagnated, and overall survival has increased by only 5% over the past 20 years. An epidemiological study showed that tobacco smoking [4–6], alcohol consumption [7, 8] and prevalence of human papilloma virus (HPV) [9] are the major risk factors. In addition, despite deep understanding of surgical techniques, adjuvant therapy, and molecular mechanisms of pathogenesis, the prognosis for patients with advanced cancer or the elderly remains poor [10, 11]. Slightly more than half of the patients were diagnosed with regional or metastatic disease, especially a propensity for lymph node metastasis at diagnosis [12, 13]. Lymph node metastasis has the most significant impact on survival, reducing survival by 50% [11]. Independent of lymph node metastasis, the occurrence of extracapsular spread (ECS) heralds a worse failure rate for regional and distant metastases [14]. The exact molecular mechanism of the pathogenesis of OSCC is not precise yet. It is unmet to understand the molecular mechanism of oral cancer metastasis to develop an effective clinical strategy for therapy of oral cancer.

Increasing evidence indicates that epithelial-mesenchymal transition (EMT) plays a crucial role in the metastasis of cancer [15–19]. Through EMT, epithelial cells losing cell polarity cause morphological alterations and acquire higher interstitial cell phenotypes, such as migration and invasion [20, 21]. EMT involves a series of molecular regulation, including the down-regulation of epithelial cell markers (E-cadherin), transmembrane protein (N-cadherin), transcription factors (Snail, Slug, Zeb-1) [22, 23]. EMT is an important biological process of malignant cell migration and invasion of epithelial origin.

The human Obg-like ATPase 1 (OLA1) is a 45 kDa protein in size and is widely present in the cytoplasm and expressed in most tissues [24]. OLA1 belongs to the YchF subfamily of the Obg-like GTPase family. It has recently been found that YchF homologous proteins have higher binding and hydrolysis efficiencies to ATP than that to GTP [25]. Structural analysis suggests that it is a regulatory protein binding to its downstream effector and exerting its regulatory function through its conformational transition [26]. OLA1 is essential for normal mammalian development and plays a vital role in regulating cell proliferation, partially by inhibiting p21 and organogenesis [27]. Chen et al. showed that OLA1 might be a novel translational GTPase that plays an important role in translation and cell survival as well as cancer development [28]. Zhang et al. showed that OLA1 may play an essential role in promoting breast cancer metastasis [29]. While, Jeyabal et al. reported that OLA1 could inhibit breast cancer cell adhesion and spread in breast cancer [30]. Knockdown of OLA1 in lung adenocarcinoma cells can attenuate the TGF-β-induced EMT process and restore E-cadherin expression [31]. The results of OLA1 expression and functions analysis in metastasis were from different cancer types, which may be the reason for the inconsistency. In summary, the exact regulatory role of OLA1 in cancer is still unknown. Our data in this study showed that OLA1 suppressed oral squamous cell metastasis by inhibition of SMAD2-EMT pathway, suggesting that OLA1 may be developed as a therapeutic target for oral metastasis.

## Results

### The endogenous level of OLA1 in OSCC and oral cell lines

**Fig. 1:**
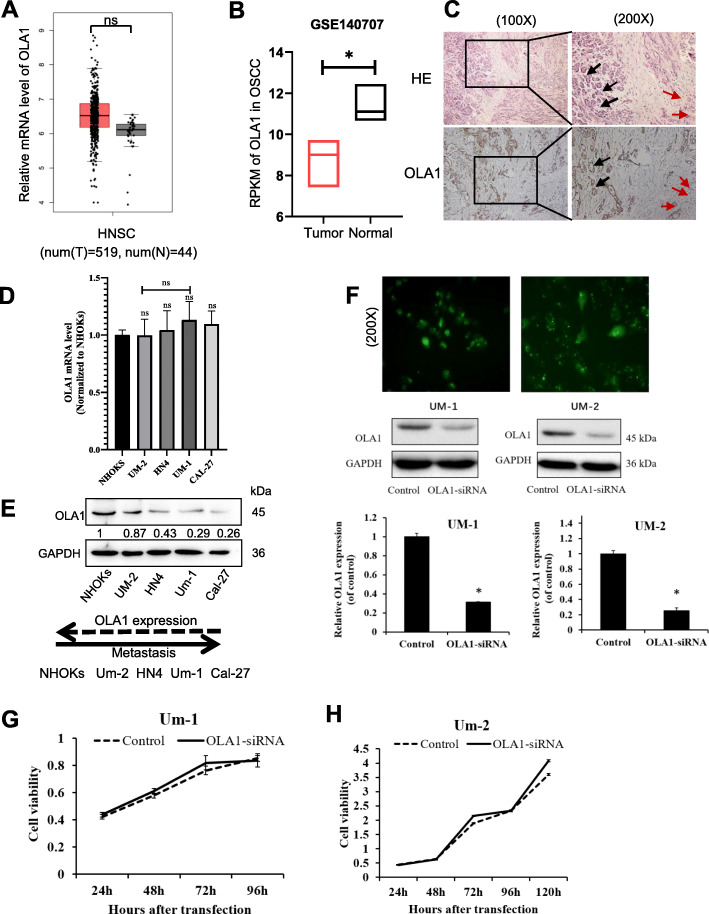
The endogenous level of OLA1 in OSCC and oral cell lines. **a** The average expression level of OLA1 in patients HNSC in TCGA and GTEx oral cancer dataset. T = Tumor, N = Normal, num = Numbers. **b** OLA1 RPKM in OSCC from GEO140707, *N* = 3 paried tissues. **c** OLA1 expression in clinical OSCC (Red arrow) and the adjacent tissues (Black arrow) *n* = 5. mRNA (**d**) and protein (**e**) level in five oral cell lines. **f** OLA1 expression in OLA1-siRNA cells. Top panel: Fluorescent photos of cells transfected with OLA1 siRNA for 24 h. Middle panel: OLA1 protein expression in indicated cells. Bottom panel: OLA1 mRNA expression in indicated cells. **g** and **h** Cell proliferation curve was drawn in indicated cells. vs. Control, *P < 0.05, ns = no significance

To understand the endogenous levels of OLA1 in oral normal and its paired tumor tissues, the OLA1 RNA sequencing data from Gene Expression Profiling Interactive Analysis (GEPIA) was analyzed [32]. We found that OLA1 is highly expressed in most of cancer, and have no difference between Head and Neck squamous cell carcinoma (HNSC) and its paired normal tissues (Figs. 1a, S1). To further investigate the OLA1 expression in OSCC, we analyzed gene expression omnibus (GEO) Datasets (GSE140707) with three tumorous and adjacent tissues from OSCC sufferers and found that OLA1 mRNA was downregulated in tumors (Fig. 1b). Immunohistochemical analysis in clinical oral tumor tissues further proved that OLA1 expression was also lower in tumors than the adjacent tissues (Fig. 1c*).* In order to investigate the role of OLA1 in oral cancer cells, five oral squamous cell lines were chosen to detect the endogenous level of OLA1. Our results also validated that OLA1 mRNA had no significant difference in five OSCC cell lines (Fig. 1d). We speculated that OLA1 might undergo post-translational modification. Therefore, we performed *SUMO (*small ubiquitin-related modifier) modification, but the results were negative*.* Interestingly*,* the endogenous level of the OLA1 protein in oral cancer cell lines was also significantly lower than that in normal oral cells, as shown in Fig. 1e. To understand whether OLA1 may be associated with oral cancer metastasis, the endogenous level of OLA1 in metastatic oral cancer cell line was analyzed. We found that OLA1 expression in metastasis cell line UM-1 was significantly lower than the carcinoma in situ cell line UM-2, suggesting a negative role OLA1 playing in oral cancer metastasis. To study the effect of OLA1 on the proliferation of oral cancer cells, silenced OLA1 assays were performed (Fig. 1f) and found that there was no significant effect observed on oral cancer cell proliferation (Fig. 1g, h), which is consistent with another report [28].

### Dysregulation of OLA1 affected the ability of metastasis in oral cancer cells

**Fig. 2:**
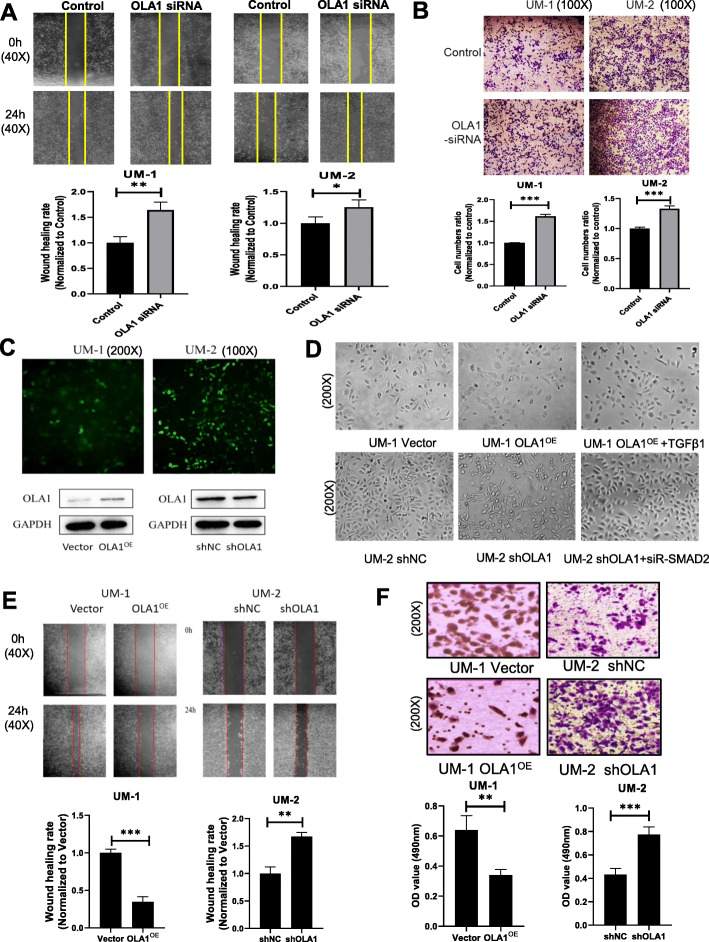
Dysregulation of OLA1 affected the ability of metastasis in oral cancer cells. **a** Wound healing assay to examine the effect of down regulating OLA1 on UM-1 and UM-2 cells (*n* = 3). **b** Transwell assay to investigate the impact of downregulating OLA1 on metastatic ability in UM-1 and UM-2 cells (*n* = 3). **c** Construction of stable cell lines with UM-1 (YFP-OLA1^OE^) plasmid or lentiviral vector in UM-2 (GFP-shOLA1). Top panel: Fluorescent photos of indicated cells. Bottom panel: OLA1 expression in relatived cells. **d** Morphology of cells (200X) after OLA1 plasmid transfection or TGFβ1 (1 ng/mL) induction. **e** Wound healing assay to examine the effect of upregulating OLA1 on UM-1 and downregulating OLA1 on UM-2 cells (*n* = 3). **f** Transwell assay to examine the effect of upregulating OLA1 on UM-1 and downregulating OLA1 on UM-2 cells (*n* = 3). vs. Control, *P < 0.05, **P < 0.01, ***P < 0.001

To determine whether OLA1 can regulate the strength of metastasis in oral cancer cells, OLA1 activity was silenced by small interfering OLA1 RNA (siR-OLA1). We found that the wound healing ability of oral cancer cells was much higher in OLA1 silenced oral cells as compared to control (Fig. 2a). The metastatic ability of oral cancer cells was also much higher in the OLA1 silenced oral cancer cells than the control cells (Fig. 2b). These data indicated that knocked-down OLA1 in UM-1 and UM-2 enhanced cell migrative ability. To further characterize the regulatory role of OLA1 in oral cancer metastasis, two oral cancer cell lines were established with either OLA1-overexpressed (OLA1^OE^) in UM-1, or OLA1 knocked down in UM-2 (shOLA1) (Fig. 2c). UM-1 OLA1^OE^ cells showed a glomerate growth morphologically, while UM-2 shOLA1 cells showed an elongated fibroblast-like morphology (Fig. 2d). This phenomenon was coincident with the initial stage of the EMT process. Invasion and metastasis of oral cancer cells were also evaluated by Transwell and wound healing assays, respectively. The wound healing rate in the UM-1 OLA1^OE^ cells was slower than control (Fig. 2e), and the numbers of the metastatic OLA1^OE^ cells were less than the Vector cells (Fig. 2f). The results in the UM-2 shOLA1 cells showed the opposite way to that in the UM-1 OLA1^OE^ cells. These data suggested that OLA1 might play a negative role in the metastasis of oral cancer.

### OLA1 regulates oral cancer metastasis through TGFβ/Smad2/Smad4 mediated EMT pathway

**Fig. 3:**
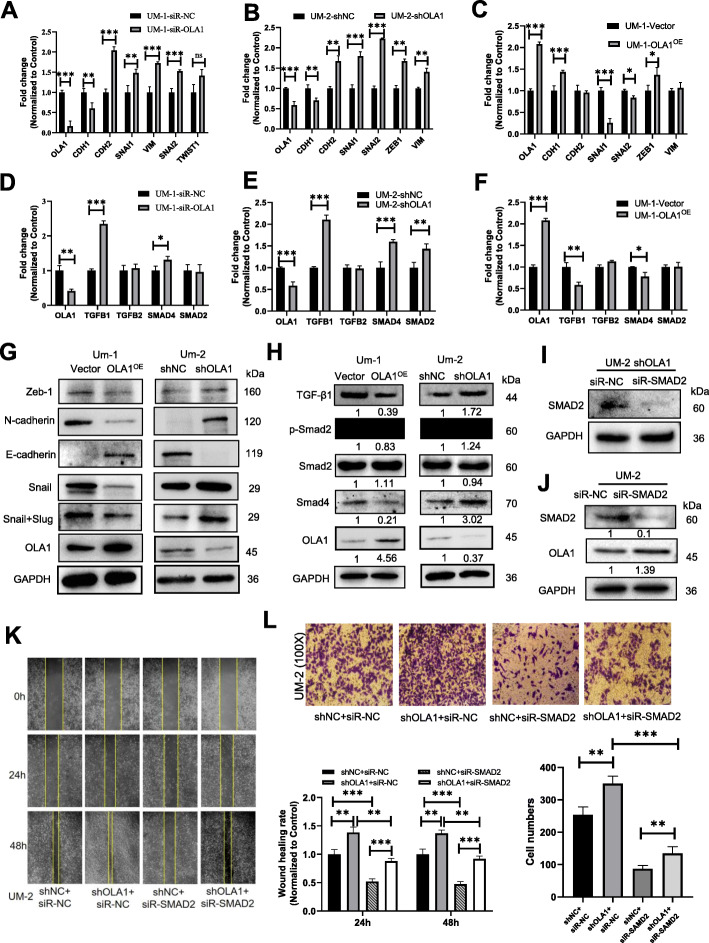
OLA1 regulates oral cancer metastasis through Smad2/Smad4 mediated EMT pathway. **a** mRNA fold change level of EMT markers between UM-1 siR-OLA1 cell and siR-NC cell. mRNA fold change level of EMT marker in UM-2 OLA1 knock down cells (**b**) and UM-1 OLA1 overexpression cells (**c**). **d** mRNA fold change level of TGFβ/SMDA2 pathway markers between UM-1 siR-OLA1 cells and siR-NC cells. mRNA fold change level of TGFβ/SMDA2 pathway markers in UM-2 OLA1 knock down cells (**e**) and UM-1 OLA1 overexpression cells (**f**). **g** Protein level of EMT marker in UM-1 OLA1 overexpression cells and UM-2 OLA1 knock down cells. **h** Protein level of TGFβ/SMDA2 pathway markers in UM-1 OLA1 overexpression cells and UM-2 OLA1 knock down cells. **i** Western blotting assay showed SMAD2 was silenced by siR-SMAD2 in UM-2 shOLA1 cells. **j** Western blotting assay showed that silenced SMAD2 decreased OLA1 expression in UM-2 cells. **k** Wound healing assay showed SMAD2 silenced could rescue enhanced metastasis induced by knocking down OLA1 in UM-2 cells (*n* = 3). **l** Transwell assay to examine the effect of OLA1 on metastasis by smad2 in UM-2 cells (*n* = 3). vs. Control, *P < 0.05, **P < 0.01, ***P < 0.001

EMT is a biological transformation process that can make polarized epithelial cells gradually present a mesenchymal cell phenotype, which is manifested in enhanced migration ability, invasiveness, and enhanced anti-apoptotic ability. To understand the molecular mechanism underlying OLA1-mediated metastasis of oral cancer cells, biomarkers in the EMT process were analyzed. The results showed that decreased expression of *OLA1* in the UM-1 siR-OLA1 or UM-2 shOLA1 cells increased the expression of CDH2 (N-cadherin), SNAI1 (Snail), SNAI2 (Slug), and VIM (**P < 0.01, ***P < 0.001), and decremental CDH1 (E-cadherin) (Fig. 3a, b). On the other hand, expression of SNAI1(Snail) and SNAI2 (Slug), was significantly decreased in UM-1-OLA1^OE^ cells, and increased CDH1 (*P < 0.05, ***P < 0.001) (Fig. 3c). Western Blotting assay showed that escalated OLA1 expression in UM-1 OLA1^OE^ cells decreased N-cadherin, Snail, Slug, and incremental E-cadherin. While in UM-2 shOLA1 cell had the contrary results (Fig. 3g), suggesting that OLA1 might regulate EMT process negatively. To further investigate the molecular mechanisms of OLA1 in the regulation of EMT resulting in metastasis of OSCC, biomarkers in TGF-β/Smad pathway including TGFB1, TGFB2, SMAD2 and SMAD4 were characterized in the following cell lines UM-1, UM-1-siR-OLA1,UM-1-OLA1^OE^, UM-2 and UM-2-shOLA1 cells. The results showed that *OLA1* knockdown increased *TGFB1*, *SMAD2* and *SMAD4* expression (Fig. 3d, e). Inversely, *OLA1* overexpression decreased *TGFB1* and *SMAD4* expression (Fig. 3f). Western Blotting verified the results of mRNA level of TGF/SMAD axis markers. Meanwhile, knocking down OLA1 could promote the phosphorylation level of SMAD2 to active TGFβ/SMAD pathway, indicating that TGF-β/Smad was inhibited in UM-1-OLA1^OE^ cells respectively (*P* < 0.05) (Fig. 3h). In order to further prove that OLA1 regulates the TGFβ/SMAD2 pathway, cellular morphology was obtained after treated with TGFβ(1 ng/mL) in UM-1 OLA1^OE^ cells or transfected siR-SMAD2 in UM-2 shOLA1 cells. TGFβ reactivated the mesenchymal phenotype of UM-1 OLA1OE cells, while silencing SMDA2 reversed the mesenchymal phenotype of UM-2 shOLA1 cells (Fig. 2d). Furtherly, UM-2 shOLA1 siR-SMAD2 cells were constructed to study the role of SMAD2 in OLA1 involvement in tumor migration. Western Blotting assay showed that we successfully silenced SMAD2 in UM-2 shOLA1 cells (Fig. 3i). Western blotting assay showed that silenced SMAD2 increased OLA1 expression in UM-2 cells (Fig. 3j). Wound healing test showed that SMAD2 silencing could rescue enhanced metastasis induced by knocking down OLA1 in UM-2 cells (Fig. 3k). Similarly, Transwell assay showed that, the number of migrating cells was reinhibited again after silenced SMAD2 in UM-2 shNC or shOLA1 cells, and back to the UM-2 shNC level or even lower (Fig. 3l). These results indicated that OLA1 overexpression weakened the EMT phenotype through the inhibition of the TGF-β/SMAD2/SMAD4 pathway in oral cancer cell lines.

### Reduced OLA1 expression is related to paclitaxel-induced cancer cell metastasis

**Fig. 4:**
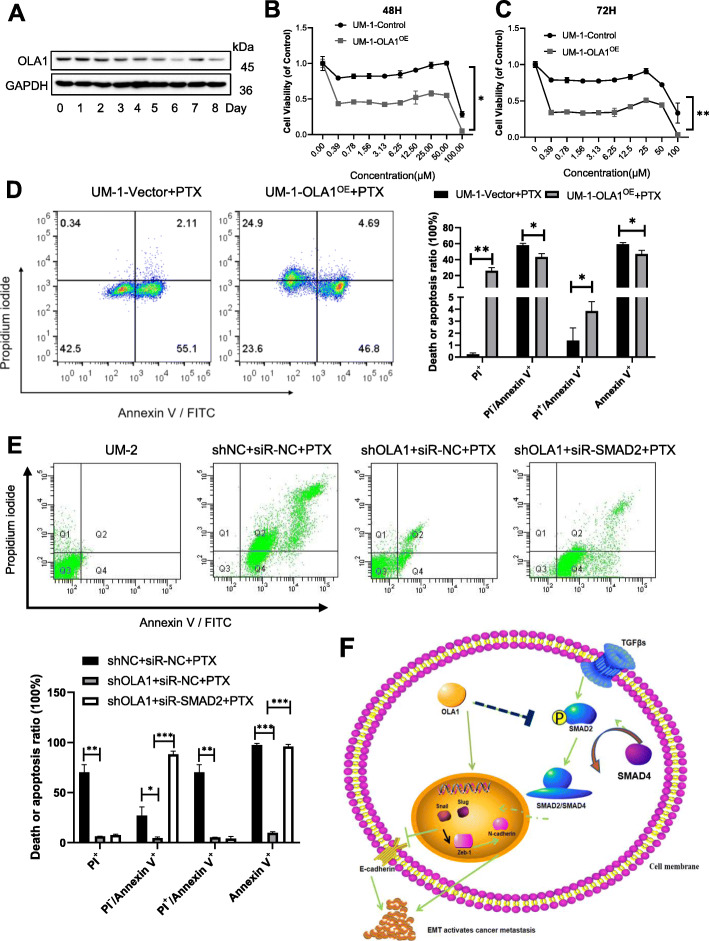
Reduced OLA1 expression is related to paclitaxel-induced cancer cell metastasis. **a** The OLA1 expression in UM-1 cell treated with 100 nM PTX for 0–8 days. **b** and **c** Cell viability assay showed that OLA1 overexpression enhanced the PTX sensitivity in UM-1 cells (*n* = 3). **d** Cell flow cytometry was used to determine the effect of overexpression of OLA1 on paclitaxel-induced (20 μM) cell necrosis and apoptosis (*n* = 2). **e** Cell flow cytometry showed the effect of OLA1 on paclitaxel-induced (20 μM) cell necrosis and apoptosis through SMAD2 (*n* = 2). **f** Mechanism of OLA1 inhibits metastasis of OSCC. vs. Control, *P < 0.05, **P < 0.01, ***P < 0.001

It has been reported in the literature that EMT transformation of cells does not necessarily lead to the occurrence of metastasis, but it does affect the chemoresistance of tumors [33]. This article also investigated whether the OLA1 medicated EMT process leading to tumor metastasis is related to tumor drug resistance. Some chemotherapeutic drugs, while killing cancer cells, also promote the metastasis of cancer cells [34–36]. To characterize the regulatory effect of OLA1 on the oral cancer metastasis induced by paclitaxel (PTX), UM-1 cells were treated with PTX (100 nM) in a time course of 0–8 days. We found that the increased concentration of the drug suppressed the expression of OLA1 (Fig. 4a). Besides, overexpression of OLA1 can increase sensitivity to PTX in UM-1 cells (Fig. 4b, c). These evidences indicated that OLA1 may increase the sensitivity of cells to drugs by reducing the migration of oral cancer cells, thus playing an anti-tumor role. Flow cytometry was used to detect the necrosis and apoptosis of cells after incubation with 20 μM PTX for 24 h. UM-1-OLA1^OE^ cells increased the late apoptosis rate of the cells and the overall mortality rate after treated with PTX. Some of the early apoptotic cells turned to necrosis instead of late apoptosis (Fig. 4d). It showed that OLA1 could increase the sensitivity of oral cancer cells to PTX. The results are consistent with the MTT experiment. Similarly, Knockdown of OLA1 greatly decreased the apoptosis of UM-2 cells by PTX. However, the continued silence of SMAD2 promoted the killing of UM-2 shOLA1 cells by PTX (Fig. 4e). These results indicate that OLA1 enhances the sensitivity of cells to drugs by inhibiting the TGF/Smad axis.

### Mechanism of OLA1 inhibits metastasis of OSCC

OLA1 is a metabolic enzyme that has both ATPase and GTPase activity, and it participates in many important physiological processes. In this article, we found that OLA1 inhibits the metastasis of OSCC cells through the TGFβ/SMAD2/SMAD4 axis (Fig. 4f). Briefly, OLA1 inhibits the expression of TGFβ1, thereby inhibiting the phosphorylation of SMAD2, resulting in a decrease in the expression of SMAD4, inhibiting the expression of Snail and Slug, the important transcription factors of EMT, increasing E-cadherin, and achieving inhibition of the EMT pathway, which ultimately leads to a decrease in the metastatic ability of tumor cells.

## Discussion

The expected curative rates for one-third of patients with OSCC diagnosed at stage I / II is about 80% (stage I) and 65% (stage II), respectively [37]. However, most cases of OSCC are diagnosed as an advanced disease (III or IV) with the 5-year survival rate of less than 50% [38], and a curative rate of 30% [37]. The survival rate of patients with metastatic disease is about 4 months [39]. Oral metastasis remains still a bottleneck for therapy of oral cancer. Previously, although there are a few studies showing the involvement of OLA1 in tumor metastasis, the exact role of OLA1 in oral cancer metastasis is not known yet.

Normal human oral keratinocytes (*NHOKs)* is normal oral cell line. UM-1 and UM-2 are oral cancer cell lines from the same patient, with the difference that UM-1 is a metastatic cell line, and UM-2 is an in-situ cell line. Cal-27 is squamous cell carcinoma with a lesion of the middle of the tongue, and poorly or undifferentiated*.* Our work indicated that OLA1 expression in oral cancer cells was significantly lower than that of normal oral cells, and decreased as the degree of cancer metastasis increases. When we knocked down or elevated the level of OLA1 in oral cancer cells UM-1 and UM-2, the phenotype of the cells changed significantly with a more EMT/MET (mesenchymal epithelial transition) status. It was further demonstrated by the scratch test and the Transwell experiment that OLA1 could substantially inhibit the metastasis and invasion of oral cancer.

EMT is an important mechanism of tumor metastasis. EMT is commonly found in cancer cells and provides a mechanism for transendothelial migration. In this article, we reported that OLA1 participates in the EMT process through the TGFβ/SMAD axis, which in turn affects tumor metastasis.

Blocking EMT may not directly affect cell death, but it can affect cancer cell resistance [33, 40, 41]. Although the imbalance/increase of proliferation is essential for tumor formation and growth, it is not the case for malignant tumors. For example, Snail zinc finger transcription factors trigger EMT, which confers epithelial cells migration and invasion characteristics during embryonic development and tumor progression. In addition to inducing phenotypic changes of tumor cells, Snail can also weaken the cell cycle and confer resistance to cell death induced by cell necrosis stimulating factors and pro-apoptotic signals. The resistance to cell death conferred by Snail provides a selective advantage for embryonic cells to migrate and settle in distant territories, which separates malignant cells from the primary tumor, invades and forms metastasis [42]. Elevated E-cadherin enhances the sensitivity of tumor cells to EGFR kinase inhibitors, and resistant cells are more like mesenchymal-like cells [43]. It is a well-known fact that TGFβ can induce EMT [44, 45]. There are also many reports about the involvement of TGFβ in tumor resistance [46, 47]. SMAD, as one of the important ways for TGFβ to induce EMT, has also been reported to be involved in tumor chemoresistance [48].

In addition, some chemotherapeutic drugs, while killing cancer cells, also promote the metastasis of cancer cells [34–36]. For example, Karagiannis et al. found that PTX could change tumor metastasis microenvironment and promote breast cancer metastasis [35]. Recently, research by Keklikoglou found that the commonly used chemotherapy drugs PTX and doxorubicin can promote the release of exosomes by tumors, change the microenvironment in the lung, and promote lung metastasis in breast cancer [36]. PTX, like cisplatin, 5-fluorouracil, and carboplatin, can be used as oral chemotherapy drugs [49]. We found that overexpression of OLA1 could increase the sensitivity of cells to PTX. Therefore, we speculate that OLA1 enhanced the sensitivity of cells to drugs by inhibiting the TGF/smad axis and EMT process. In order to verify our conjecture, we silenced SMAD2 expression in UM-2 shOLA1 cells, and the killing effect of PTX on the cells was investigated by a flow cytometry assay. Knockdown of OLA1 greatly decreased the apoptosis of UM-2 cells by PTX. However, the continued silence of SMAD2 promoted the killing of UM-2 shOLA1 cells by PTX. The experimental results show that OLA1 enhances the sensitivity of cells to PTX by inhibiting the process of TGF/SMAD and EMT. Therefore, we report that OLA1 can inhibit the EMT process induced by TGFβ/SMAD2, thereby increasing the drug sensitivity of oral cancer cells to PTX.

## Conclusion

We observed that OLA1 may play a negative role in the metastasis and invasion of oral cancer and this effect may be achieved through the EMT pathway, which plays a vital role in the diagnosis and treatment of clinical oral cancer. However, doubtless further investigations, including in vivo animal model studies and prospective clinical observations, are implemented. We tried to make an orthotopic oral cancer metastasis model to verify the inhibitory effect of OLA1 on oral cancer metastasis by inoculating human oral cancer metastasis cell line UM-1 in nude mice. However, we failed to make an oral cancer metastasis model in situ, which is not as simply as in subcutaneou transplantation tumor or liver metastasis model. Therefore, we plan to use OLA1^(−/−)^ systemic knockout mice to further study the function of OLA1 in inhibiting oral cancer metastasis using a drug-induced oral carcinoma in situ in the future.

## Methods

### Chemicals and reagents

DMEM-F12 medium and foetal bovine serum (FBS) were purchased from the Gibco (Invitrogen, USA). Penicillin, streptomycin and BCA protein assay kits were purchased from the Solarbio (Beijing, China). Polystyrene plate for transwell was purchased from the Corning (New York, USA). The primary antibodies were diluted 1:2000 before use, including OLA1 (Cat. #ab229090, Abcam), GAPDH (Cat. #ab9485, Abcam), GAPDH (Cat. #ab8245, Abcam), N-cadherin (Cat. #ab18203, Abcam), E-cadherin (Cat. #ab76055, Abcam), Snail (Cat. #PA5–11923, Invitrogen), Snail + Slug (Cat. # ab180714, Abcam), Zeb-1 (Cat. # ab155249 Abcam) and SMAD2 (Cat. #5339, Cell Signaling Technology), p-SMAD2 (Cat. #18338, Cell Signaling Technology), SMAD4 (Cat. #9515P, Cell Signaling Technology). TGF-beta 1 antibody (Cat. # 21898–1-AP) was purchased from ProteinTech (Wuhan, China). All the chemical compounds were analytically pure reagents.

### Cells and cell culture

The human OSCC cell lines UM-1 and UM-2 were donated by Prof. Hu (UCLA, USA). HN4, NHOKS and CAL-27 were purchased from the American Type Culture Collection (ATCC). Cells were cultured in a 1:1 mixture of Dulbecco’s modified Eagle medium and Nutrient Mixture F-12 medium with 10% FBS, 100 IU/mL penicillin and 100 μg/ml streptomycin in 37 °C, 5% CO_2_ incubator.

### OSCC tissue HE staining and immunohistochemistry

We obtained five tissues diagnosed as oral squamous cell carcinoma by pathology from Dalian Stomatological Hospital. The protocols for collection and analysis of these tissues and experiments about processing and using of tissue samples was approved by the Biology and Medical Ethics Committee of Dalian University of Technology and have obtained the patients signed informed consent. Hematoxylin-eosin (HE) staining is used to judge the location of tumor tissue, and immunohistochemistry is used to evaluate the expression of OLA1 (1:200) in tumor tissue.

### Small interfering RNA transfections

Small interfering RNA (siRNA) for OLA1 (siR-OLA1) marked with FAM Probe, small interfering RNA for SMAD2 (siR-SMAD2, Sense: 5′-GUCCCAUGAAAAGACUUAATT-3′, Anti-sense: 5′-UUAAGUCUUUUCAUGGGACTT-3′) and the control siRNA (Sense: 5′-UUCUCCGAACGUGUCACGUTT-3′, Anti-sense: 5′-ACGUGACACGUUCGGAGAATT-3′) were purchased from GenePharma (Shanghai, China). Cells seeded in a 6-well plate were transiently transfected with 100 pM siRNA with the Lipofectamine 2000 Transfection Reagent (Thermo Scientific) according to manufacturer’s instructions for 24 h.

### Establishment of the stable OLA1 knockdown UM-2 cell lines

Small hairpin RNA (shRNA) lentiviral used for stable silencing of OLA1 (shOLA1) and the control non-targeting plasmid (shNC) were purchased from GenePharma (Shanghai, China) by inserting the following short-hairpin sequences into the pGLV3/H1/GFP/Puro vector: 5′-CCGGGAGGAAATGATTGGGCCCATTCTCGAGAATGGGCCCAATCATTTCCTCTTTTTTG-3′ for sh-OLA1 and 5′-CCGGCAACAAGATGAAGAGCACCAACTCGAGTTGGTGCTCTTCATCTTGTTGTTTTTG-3′ for Small hairpin (sh) control. Small hairpin RNA (shRNA) transfections and protocol were followed the recommendations by GenePharma (Shanghai, China). The shNC and shOLA1 vectors were transfected into UM-2 cells. The knockdown efficiency of the target gene was verified by fluorescence imaging, qRT-PCR and western blot analysis.

### Construction of OLA1 stable overexpression cell line

OLA1-YFP plasmid (pdEYFP-N1gen plasmid with a C-terminal YFP tag, OLA1^OE^) was donated by Prof. Zhengzheng Shi. UM-1 Cells in 6-well plates were transfected with the related plasmid (4 μg DNA/well) or the control plasmid using Lipofectamine 2000 (8 μl/well, Invitrogen). The overexpression efficiency of the target gene was verified by qRT-PCR and western blot analysis.

### Cell viability assay

To investigate the effect of knocking down OLA1 on the proliferation ability of UM-1 and UM-2 cells, the growth curves of cells at 24 h, 48 h, 72 h and 96 h after transfection with siR-OLA1 were drawn. MTT(3-(4,5-dimethyl-2-thiazolyl)-2,5-diphenyl-2-H-tetrazolium bromide) assay was also used to determine the viability of the treated cells. 6 × 10^3^ cells were seeded onto 96-well plates and incubated overnight at 37 °C. PTX at different concentrations (0, 0.39, 0.78, 1.56, 3.13, 6.25, 12.5, 25.0, 50.0, 100.0 μM) were added to each well with different incubation times. The absorbance was measured at 490 nm by the microplate reader. Six replicate wells were included in each analysis, and at least three independent experiments were conducted.

### qRT-PCR analysis

Total RNA was extracted from the cells using TRIzol (Cat. #15596026, Invitrogen, CA, USA), and the concentration and quality were determined by a microplate reader (DU730, Beckman, CA, USA). The nucleotides were reverse-transcribed into cDNA according to the instructions of the PrimeScript RT Reagent Kit (Cat. #RR037A, Takara, Japan). After amplification and dilution, the PCR assay was performed on the LightCycler480 II (Roche, USA). Gene primer as follows: GAPDH (glyceraldehyde 3-phosphate dehydrogenase): Forward primer: 5-CATGAGAAGTATGACAACAGCCT, Reverse primer: 5-AGTCCTTCCACGATACCAAAGT; OLA1: Forward primer: 5-TGGACAAGTATGACCCAGGT, Reverse primer: 5-GCTGCAAACCCAGCCTTAATG; CDH1: Forward primer: 5-CGAGAGCTACACGTTCACGG, Reverse primer: 5-GGGTGTCGAGGGAAAAATAGG; CDH2: Forward primer: 5-AGGCTTCTGGTGAAATCGCA, Reverse primer: 5-TGCAGTTGCTAAACTTCACATTG; SNAI1: Forward primer: 5-ACTGCAACAAGGAATACCTCAG, Reverse primer: 5-GCACTGGTACTTCTTGACATCTG;

SNAI2: Forward primer: 5-CACACGGGGGAGAAGCCTTT, Reverse primer: 5-ATTGCGTCACTCAGTGTGCT; VIM: Forward primer: 5-AGGCAAAGCAGGAGTCCACTGA, Reverse primer: 5-ATCTGGCGTTCCAGGGACTCAT; TGFB1: Forward primer: 5-GCAAGTGGACATCAACGGGT, Reverse primer: 5-TCCGTGGAGCTGAAGCAATA; TGFB2: Forward primer: 5-GGTACCTTGATGCCATCCCGCC, Reverse primer: 5-GCACTCTGGCTTTTGGGTTCTGCA; SMAD2: Forward primer: 5-CCGACACACCGAGATCCTAAC, Reverse primer: 5-GAGGTGGCGTTTCTGGAATATAA; SMAD4: Forward primer: 5-GGTTCCTTCAAGCTGCCCTA, Reverse primer: 5-ATGTGCAACCTTGCTCTCTCA;

### Western blot analysis

Proteins were extracted from cells using 200 μL RIPA lysis buffer for 30 min at 4 °C, vortexed every 10 min, and then centrifuged at 12,000×g for 15 min. 20 μg sample was separated on 12% SDS-PAGE gels and blotted onto PVDF membranes. Bands were visualized by a Western Blotting Detection System (Bio-Rad Gel Doc XR+, USA).

### Transwell assay

Each Transwell chamber was filled with 1:5 diluted Matrigel 50 μL, and 37 °C stewed for 3 hours to solidify. The cells were cultured for 24 h in FBS-free medium, then were digested to make a 1 × 10^5^ cells density suspension (1% FBS), 200 μL was added to the Transwell chamber, 500 μL of medium (10% FBS) was added to the lower compartment of the chamber. After 24 h of culture, the upper chamber cells were wiped off, washed three times with PBS, fixed with paraformaldehyde for 30 min, stained with 0.1% crystal violet for 15 min, washed three times with PBS, and photographed after air drying. Two replicate wells were included in each analysis, six pictures were captured in each well and at least three independent experiments were conducted. Metastatic cell numbers were calculated by ImageJ software (National Institutes of Health, USA).

### Wound healing assay

When the cells growth in the 6-well plate covered about 80% of the bottom of the well, transfected siRNA (100pM) into the cells with Lipo2000 and MEM medium, and replace the MEM medium with DMEM-F12 medium containing 2% FBS after 5 h. When the cells filled the entire bottom of the well, a 200 μL sterile pipette tip was used to make a wound scratch on the bottom of the well, and washed with PBS for three times to remove cell debris. Take photos of wounded scratches at 0 h, 24 h or 48 h by Inverted microscope (*Olympus*, Japan). Two replicate wells were included in each analysis, six pictures were captured in each well and at least three independent experiments were conducted. The area of wound healing was calculated using ImageJ software (National Institutes of Health, USA).

### Flow cytometry

6 × 10^5^ cells were seeded in a 6-well plate, transfected with siRNA (100pM) for 24 h, then treated with PTX (20 μM) for 24 h. Flow cytometry was used to detect cell necrosis and apoptosis. The operation steps were carried out according to the manual introduction. The brief is as follows: cells were trypsinized and centrifuged at 300 g for 5 min at 4 °C, then washed twice with pre-cooled PBS. Staining cells with binding buffer containing Annexin V-FITC and PI for 10 min, then detected by a Flow cytometer (Attune NxT, Life, USA) and Flow cytometer (BD, USA).

### Statistical analysis

All data were expressed as the Mean ± SD. All statistic results were carried by GraphPad Prism8. A two-sided tail non-paired Student’s t test or multi T-test was used to compare differences between the treated group and the control group. A value with *P* < 0.05 was considered significant.

## Supplementary information

**Additional file 1: Figure S1.** OLA1 expression profile across all tumor samples and paired normal tissues.

**Additional file 2.** Original gel scan.

## Data Availability

All data generated or analyzed during this study are included in this published article. The datasets used and/or analyzed during the current study are available from the corresponding author on reasonable request.

## Abbreviations

OLA1: Obg-like ATPase 1
OSCC: Oral squamous cell carcinoma
HNSC: Head and neck squamous cell cancer
NHOKs: Normal human oral keratinocytes
EGFR: Epidermal growth factor receptor
OCC: Oral cell carcinoma
HPV: Human papilloma virus
ECS: Extracapsular spread
EMT: Epithelial-mesenchymal transition
MET: Mesenchymal epithelial transition
*GEPIA*: *Gene Expression Profiling Interactive Analysis*
*GEO*: *Gene expression omnibus*
SUMO: Small ubiquitin-related modifier
siRNA: Small interfering RNA
siR-OLA1: Small interfering OLA1 RNA
shNC: Small hairpin RNA normal control
shOLA1: Small hairpin OLA1 RNA
OLA1^OE^: OLA1 overexpression
siR-SMAD2: Small interfering SMAD2 RNA
*PTX*: *Paclitaxel*
*FBS*: Foetal bovine serum
HE staining: Hematoxylin-eosin staining
*MTT*: 3-(4,5-dimethyl-2-thiazolyl)-2,5-diphenyl-2-H-tetrazolium bromide
