# Supplementary material for: Obg-like ATPase 1 inhibited oral carcinoma cell metastasis through TGFβ/SMAD2 axis in vitro
Source: BMC Mol Cell Biol. 2020 Sep 14;21:65. doi: 10.1186/s12860-020-00311-z (PMC7489017; doi:10.1186/s12860-020-00311-z)
Supplement: Supplementary file 1 — Additional file 1: Figure S1. OLA1 expression profile across all tumor samples and paired normal tissues. [file 12860_2020_311_MOESM1_ESM.pdf]

Additional file 2:

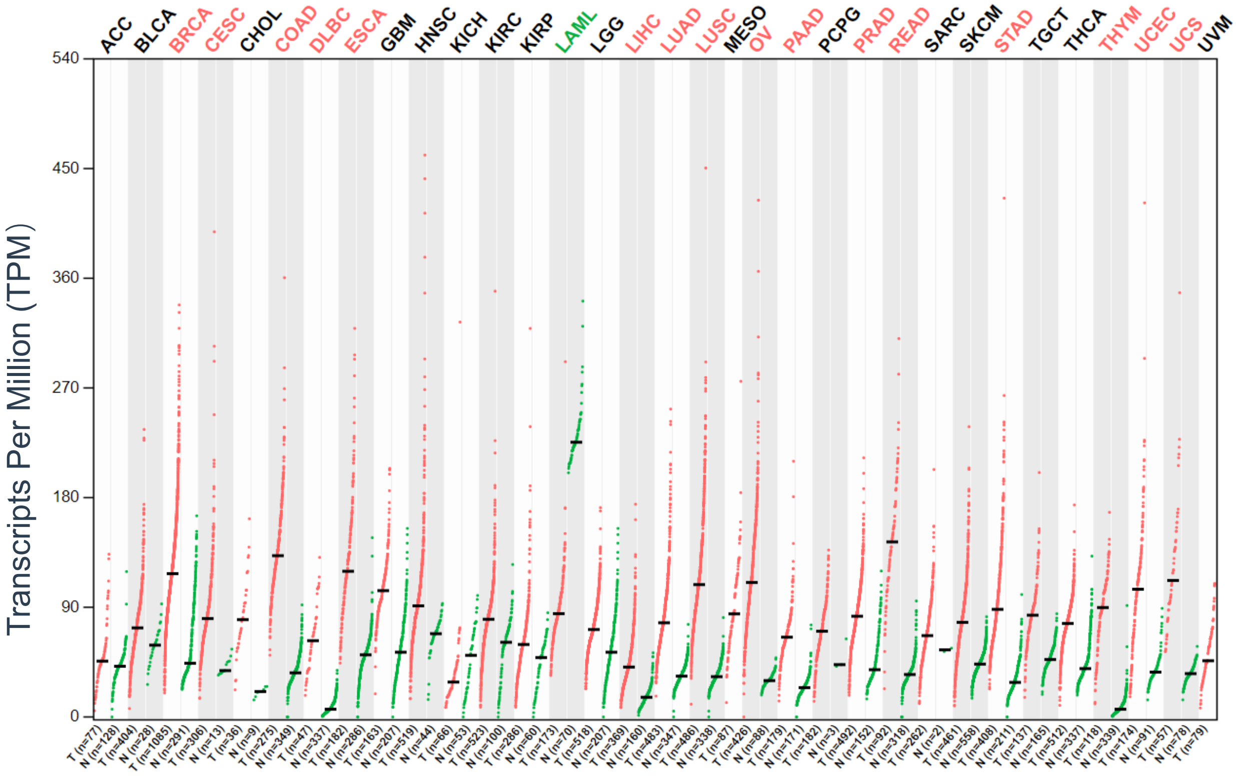

Fig. S1 OLA1 expression profile across all tumor samples and paired normal tissues. Each dots represent expression of samples. The different colors of the tumor abbreviation font represent the expression of OLA1 in the tumor relative to normal tissues. Red represents high expression in tumor tissue, green represents low expression in tumor tissue, black represents no change.

TCGA Datastes

Tumor abbreviation	Detail
ACC	Adrenocortical carcinoma
BLCA	Bladder Urothelial Carcinoma
BRCA	Breast invasive carcinoma
CESC	Cervical squamous cell carcinoma and endocervical adenocarcinoma
CHOL	Cholangio carcinoma
COAD	Colon adenocarcinoma
DLBC	Lymphoid Neoplasm Diffuse Large B-cell Lymphoma
ESCA	Esophageal carcinoma
GBM	Glioblastoma multiforme
HNSC	Head and Neck squamous cell carcinoma
KICH	Kidney Chromophobe
KIRC	Kidney renal clear cell carcinoma
KIRP	Kidney renal papillary cell carcinoma
LAML	Acute Myeloid Leukemia
LGG	Brain Lower Grade Glioma
LIHC	Liver hepatocellular carcinoma
LUAD	Lung adenocarcinoma
LUSC	Lung squamous cell carcinoma
MESO	Mesothelioma
OV	Ovarian serous cystadenocarcinoma
PAAD	Pancreatic adenocarcinoma
PCPG	Pheochromocytoma and Paraganglioma
PRAD	Prostate adenocarcinoma
READ	Rectum adenocarcinoma
SARC	Sarcoma
SKCM	Skin Cutaneous Melanoma
STAD	Stomach adenocarcinoma
TGCT	Testicular Germ Cell Tumors
THCA	Thyroid carcinoma
THYM	Thymoma
UCEC	Uterine Corpus Endometrial Carcinoma
UCS	Uterine Carcinosarcoma
UVM	Uveal Melanoma
