# Supplementary material for: Obg-like ATPase 1 inhibited oral carcinoma cell metastasis through TGFβ/SMAD2 axis in vitro
Source: BMC Mol Cell Biol. 2020 Sep 14;21:65. doi: 10.1186/s12860-020-00311-z (PMC7489017; doi:10.1186/s12860-020-00311-z)

Additional file 2: Original gel scan

Fig. S1. Uncropped images of immunoblots for Fig. 1E.

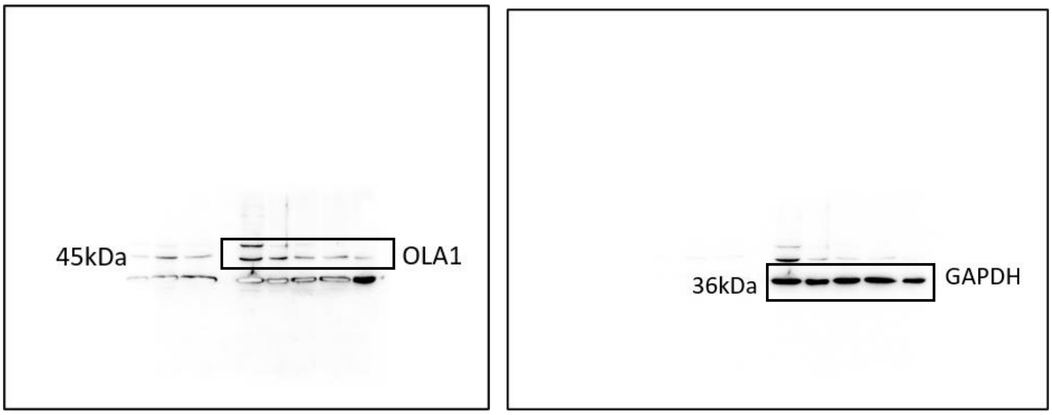

Fig. S2. Uncropped images of immunoblots for Fig. 3G.

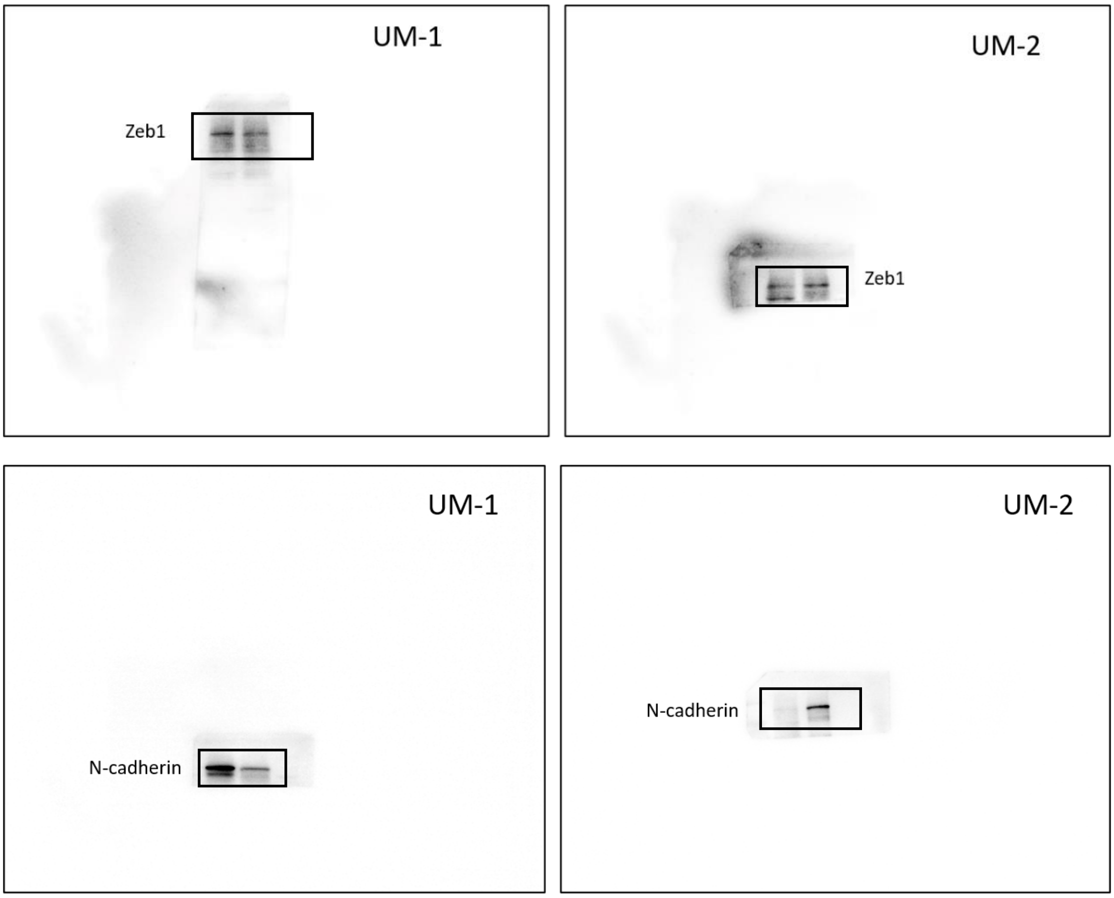

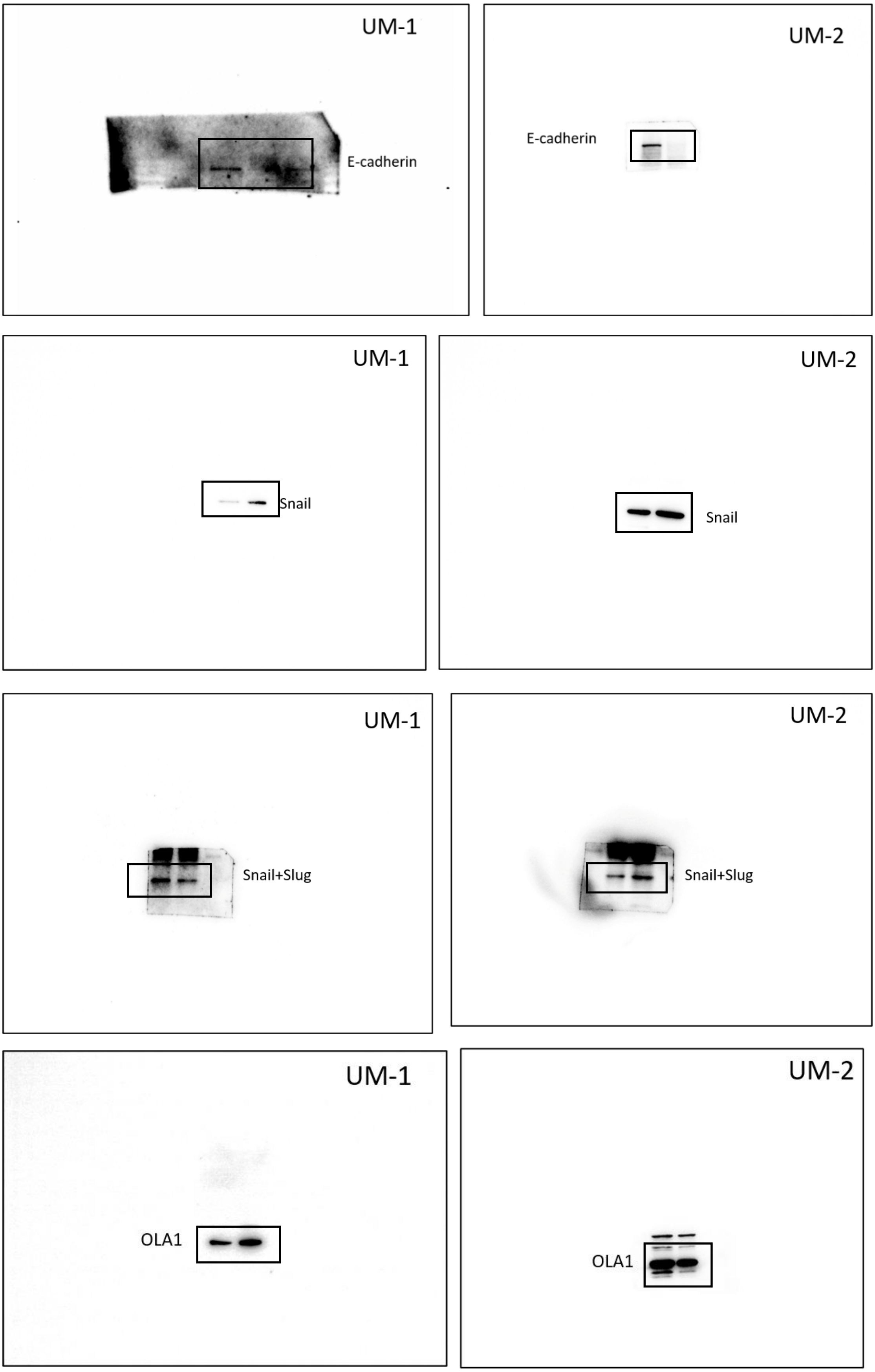
UM-1
E-cadherin
UM-2
E-cadherin
UM-1
Snail
UM-2
Snail
UM-1
Snail+Slug
UM-2
Snail+Slug
UM-1
OLA1
UM-2
OLA1

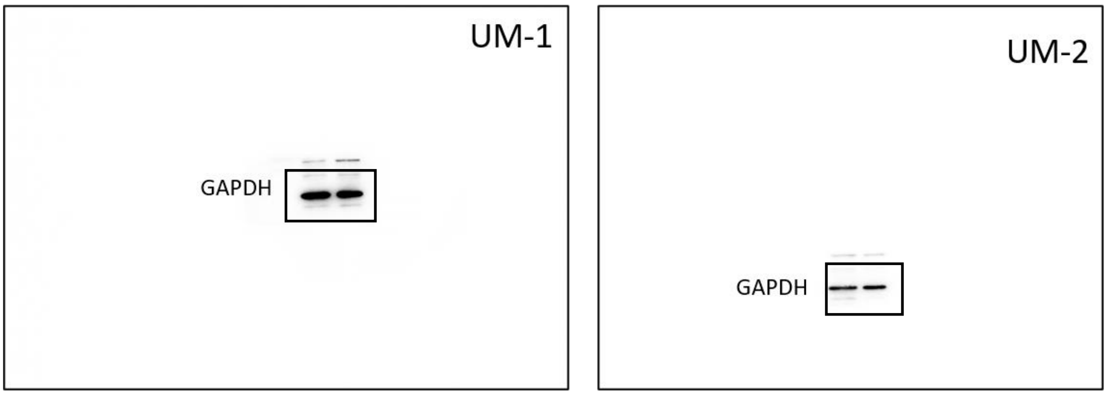

Fig. S3. Uncropped images of immunoblots for Fig. 3H.

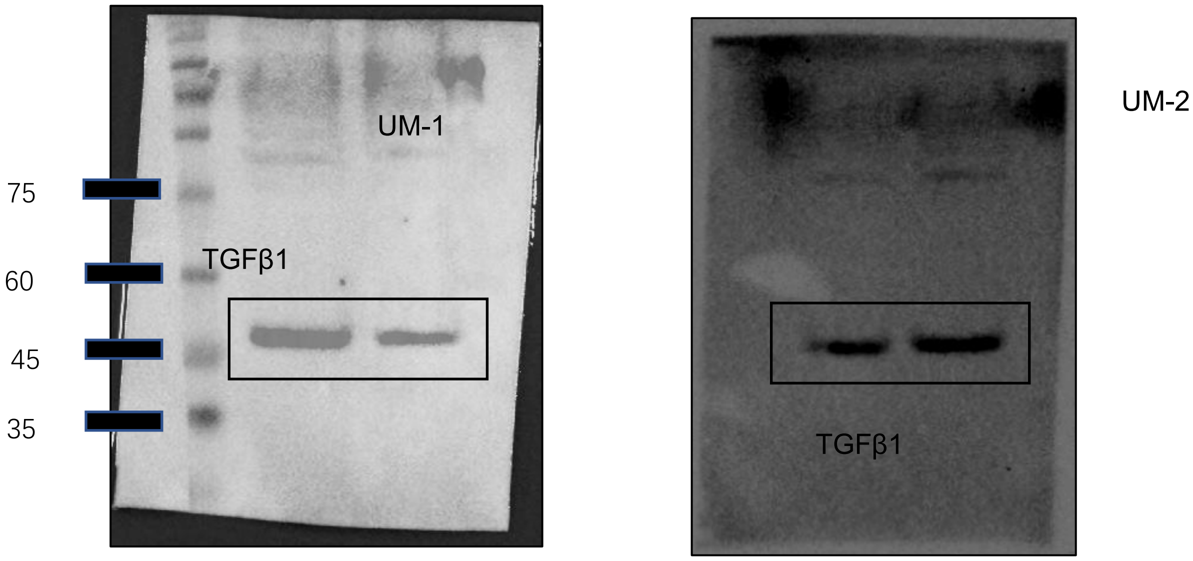

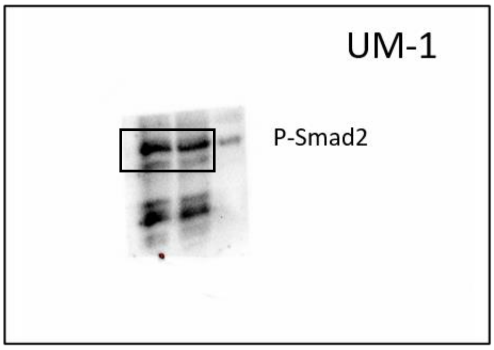
UM-1
P-Smad2

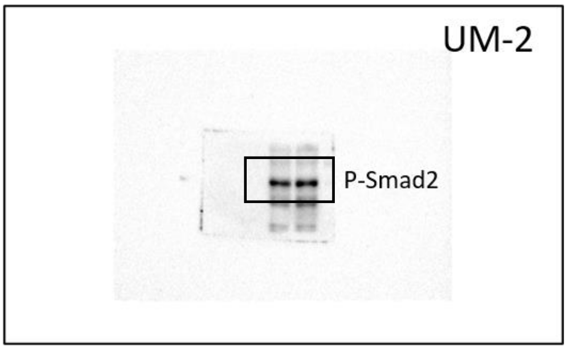
UM-2
P-Smad2

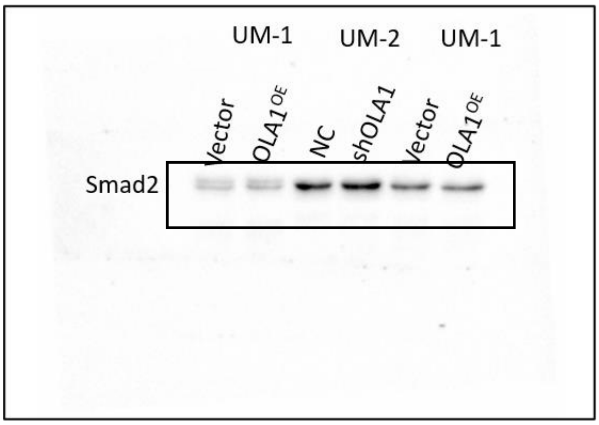
UM-1
UM-2
UM-1
Vector
OLA1OE
NC
shOLA1
Vector
OLA1OE
Smad2

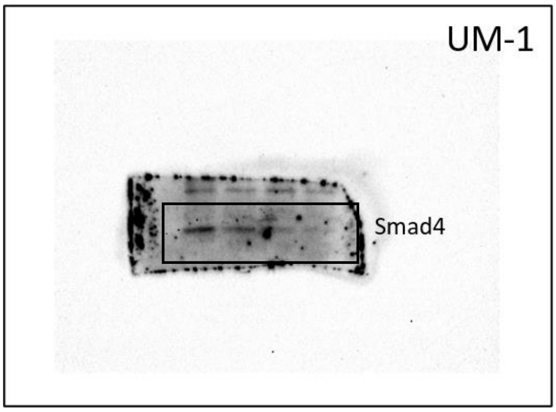
UM-1
Smad4

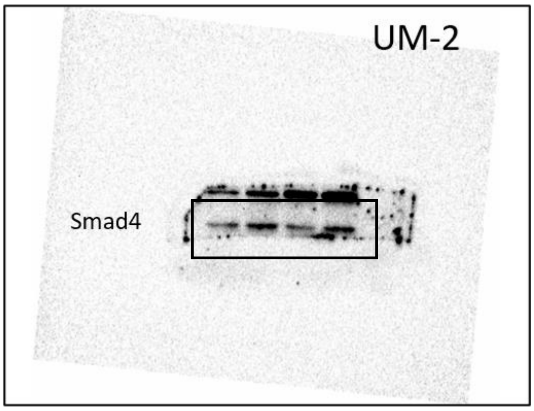
UM-2
Smad4

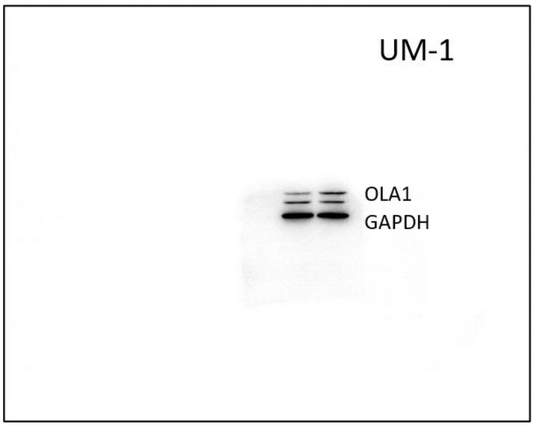
UM-1
OLA1
GAPDH

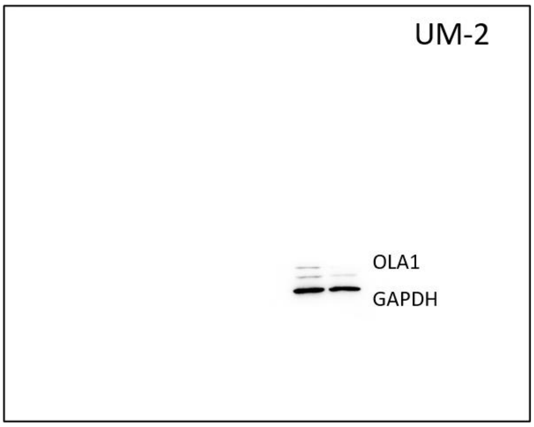
UM-2
OLA1
GAPDH

Fig. S4. Uncropped images of immunoblots for Fig. 3I.

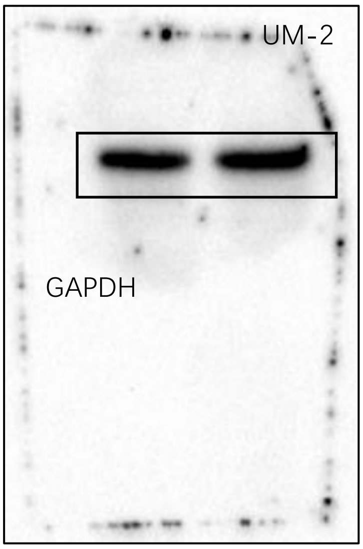

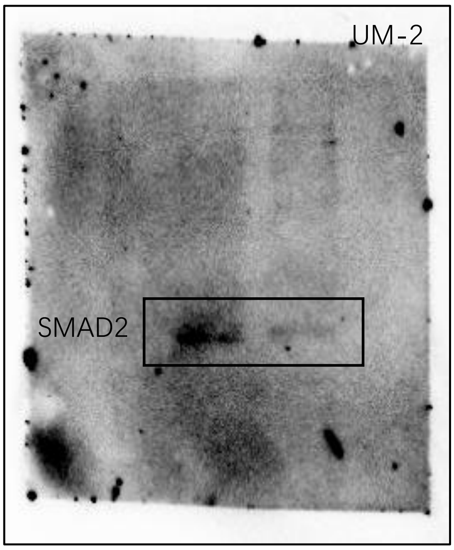

Fig. S5. Uncropped images of immunoblots for Fig. 3J.

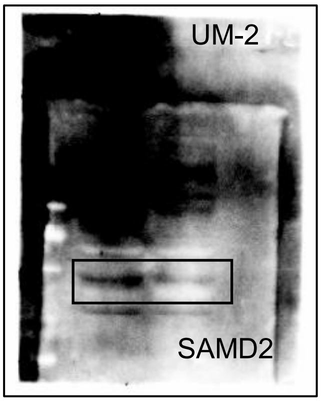

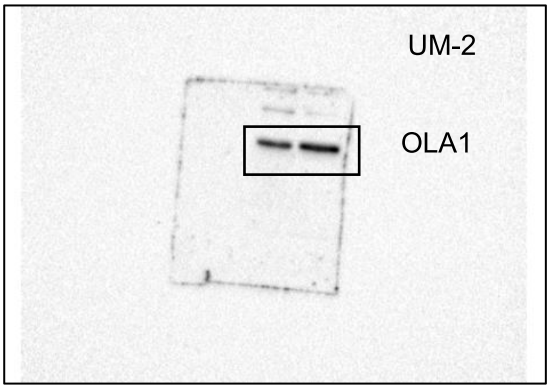

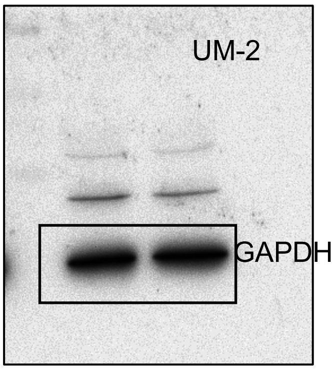

Fig. S6. Uncropped images of immunoblots for Fig. 4A.

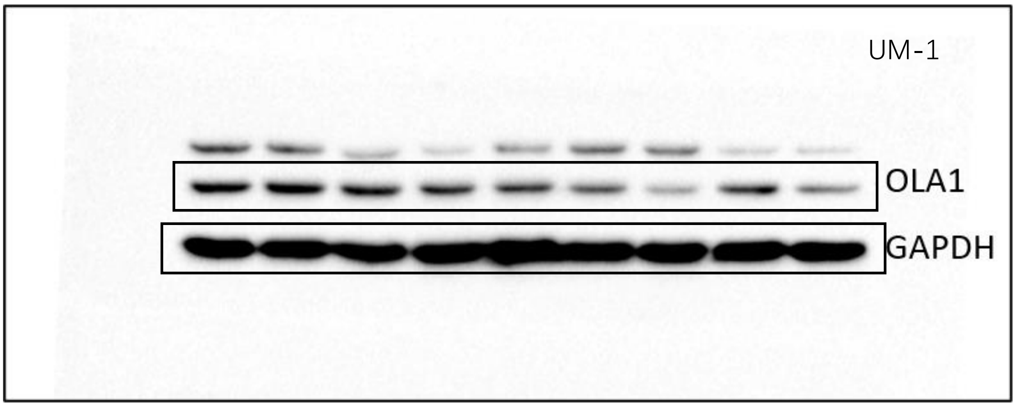

Supplement: Supplementary file 2 — Additional file 2. Original gel scan. [file 12860_2020_311_MOESM2_ESM.pdf]
